# Unraveling the evolutionary scenario of the *hobo* element in populations of *Drosophila melanogaster* and *D. simulans* in South America using the TPE repeats as markers

**DOI:** 10.1590/1678-4685-GMB-2015-0049

**Published:** 2016

**Authors:** Geovani T. Ragagnin, Larissa P. Bernardo, Elgion L. S. Loreto

**Affiliations:** 1 Curso de Ciências Biológicas, Universidade Federal de Santa Maria, Santa Maria, RS, Brazil; 2 Programa de Pós-Graduação em Biodiversidade Animal, Universidade Federal de Santa Maria, Santa Maria, RS, Brazil; 3 Departamento de Bioquímica e Biologia Molecular, Universidade Federal de Santa Maria, Santa Maria, RS, Brazil

**Keywords:** transposable elements, transposons, hAT, genomic evolution, molecular evolution

## Abstract

Transposable elements (TEs) are nucleotide sequences found in most studied genomes. These elements are highly diversified and have a large variation in nucleotide structure and mechanisms of transposition. *hobo* is a member of class II, belonging to *hAT* superfamily, described in*Drosophila* melanogaster, and it presents in its Open Reading Frame, a repetitive region encoding the amino acids threonine-proline-glutamic acid (TPE), which shows variability in the number of repeats in some regions of the world. Due to this variability some evolutionary scenarios of the *hobo* element are discussed, such as the scenario of the invasion of *hobo* element in populations of*D. melanogaster*. In the present study, we investigated 22 DNA sequences of *D. melanogaster* and seven sequences of*D. simulans*, both from South America, to check the number of repetitions of TPE, in order to clarify the evolutionary scenario of the*hobo* element in these populations. Our results showed a monomorphism in populations of both species in South America, with only three TPE repeats. Hence, we discuss and propose an evolutionary scenario of the invasion of the *hobo* element in populations of *D. melanogaster* and *D. simulans*.

Transposable Elements (TEs) are sequences of DNA that have the intrinsic ability to move in the host genome (Biémont and Vieira, 2006). This ability gives TEs a wide distribution in the genome of eukaryotes. In humans, 45% of genetic material is composed of TEs, while in *Drosophila melanogaster* about 20% and 80% of genome in maize (International Human Genome Sequencing Consortium, 2001; Meyers *et al.*, 2001; Biémont and Vieira, 2006). Furthermore, the TEs can be involved in events of Horizontal Transfer (HT), a process by which the genetic material is transferred to another who is not his or her descendant (Capy *et al.*, 1994).

TEs are considered mutagenic agents causing structural and regulatory changes in genes. Also, they promote chromosome rearrangements and epigenetic modifications (Biémont 2010; Hua-Van *et al.*, 2011). TEs show a great diversity in nucleotide sequence and structural organization and are classified based in these characteristics. The first classification was proposed by Finnegan (1989) who divided TEs into two major groups, using the transposition mechanisms and the type of intermediary in the transposition process as criteria. Class I, or retrotransposons, utilize RNA as intermediary and use a mechanism called "copy-and-paste" to replicate. Class II, or transposons, use DNA as intermediary and use a mechanism called "cut-and-paste". Wicker*et al.* (2007) proposed a new classification system, retaining the two described classes and creating new inclusive categories such as subclass, order, super-family, family and subfamily based on sequence similarities and structural relationships.

The superfamily *hAT* belongs to class II. Its name is given by the initial of the first elements described and included in this superfamily:*hobo* from *D. melanogaster*, *Ac*from maize and *TAM 3* from *Antirrhinum majus* (Warren *et al.*, 1994). These elements have characteristics in common, such as the presence of terminal inverted repeats (TIRs), generally 12 base pairs (bp) long and produce a duplication of the target site (TSDs) of eight bp. The hAT superfamily is old and their wide occurrence in higher taxonomic groups (animals, plants and fungi) suggests the results of many HT events (Rubin *et al.*, 2001).

The *hobo* element described in *D. melanogaster* by McGinnis *et al.* (1983) is observed in three different forms in the host genome: i) as a canonical and autonomous element, ii) canonical elements with internal deletion, and iii) the "relic" elements that are degenerated copies (Ortiz and Loreto, 2008). Evidence has shown that the autonomous elements as well as those sequences showing internal deletions are recent in the genomes, but the "relics" are old and probably the result of ancient invasions (Periquet *et al.*, 1994; Simmons *et al.*, 1998; Galindo *et al.*, 2001; Torres *et al.*, 2006).

*hobo* has in its Open Reading Frame (ORF) a repetitive sequence ACTCCAGAA that encodes the threonine-proline-glutamic acid amino acids (TPE) and presents variability in the number of copies of this repetitions (Calvi *et al.*, 1991; Bazin and Higuet, 1996; Souames *et al.*, 2003a, b). The majority of natural populations of *D. melanogaster* are monomorphic, with three copies of repetitions, while others are polymorphic. These are found in the center of Europe, in equatorial Africa and western South America, with differences in the number of copies, ranging from three to nine repetitions (Bazin and Higuet, 1996; Bonnivard*et al.*, 2000). A temporal and geographic distribution pattern relative to TPE copy number have been observed in the populations of *D. melanogaster* . From this pattern, some evolutionary scenarios are proposed. For example, it is suggested that the first invasion of *hobo* elements in populations of *D. melanogaster*, throughout the world, was made by elements containing three repeats (Periquet *et al.*, 1989), followed by another invasion of*hobo* elements containing five to seven repetitions of TPE (Bonnivard *et al.*, 2000; Souames *et al.*, 2003a, b). The TPE region of *hobo*elements also appears to be related to the invasive ability of the element. Comparative studies with transgenic strains of *D. melanogaster,* containing 3 or 5 TPE repeats suggests that the TPE region has a relationship with transposition or activation of *hobo* elements, such that copies of *hobo*elements containing three TPE increased the transposition activity in the host genome compared to copies containing five repetitions (Souames*et al.*, 2003a).

The *hobo* element has an interesting history in relation to this pattern. Research with old strains of *D. melanogaster* of South America (collected before 1950) showed that the canonical element was absent in these populations, while recent strains present the element. This suggests that the element has been introduced in *D. melanogaster* by HT (Periquet *et al.*, 1989; Boussy and Daniels, 1991; Pascual and Periquet, 1991). Streck *et al.* (1986) and Boussy and Daniels (1991) described the *hobo* element in *D. simulans,* and the evolutionary history of the element in this species is quite similar to the history in *D. melanogaster.* However, these authors suggest that *D. simulans* may have been the donor of the element to*D. melanogaster*, as the *hobo* element is similar between the two species, and yet it apparently was present in *D. simulans* before *D. melanogaster*.

Thus, using the TPE region of the *hobo* element as a marker, the objective of this study was to investigate the polymorphism in populations of *D. melanogaster* in South America, trying to establish a geographic distribution scenario of the element in these populations. We also analyzed the TPE regions in Brazilian populations of *D. simulans* trying to determine the monomorphic condition of three repetitions of TPE, as supposed by the invasion theory of*hobo* elements in *D. melanogaster* from *D. simulans*.

We utilized populations of *D. melanogaster* and *D. simulans* from different locations in South America and Europe (see Table 1 and Figure 2). European populations were used as a control since they are known to possess polymorphisms in the TPE region. The polymorphism analyses were made by Sanger DNA sequencing and polyacrylamide gel electrophoresis (PAGE). DNA extraction was performed in approximately 15 individuals of each population studied according to the protocol of Oliveira *et al.*(2009).

**Table 1:** Table showing the number of repetitions of TPE in populations of *D. melanogaster* and *D. simulans* in South America and the United States, including four European populations.

Population	Species	Collection site; (State / Province); country	TPE repeats	Method used to obtain the number of repetitions of TPE
1	*D. melanogaster*	Rio de Janeiro; (RJ); Brazil	3	Sequencing
2	*D. melanogaster*	Erechim; (RS); Brazil	3	Sequencing and PAGE
3	*D. melanogaster*	São José do Rio Preto; (SP); Brazil	3	PAGE
4	*D. melanogaster*	São José do Rio Preto; (SP); Brazil	3	PAGE
5	*D. melanogaster*	São José do Rio Preto; (SP); Brazil	3	Sequencing and PAGE
6	*D. melanogaster*	Santa Maria; (RS); Brazil	3	Sequencing
7	*D. melanogaster*	Porto Alegre; (RS); Brazil	3	Sequencing and PAGE
8	*D. melanogaster*	Rio Grande; (RS); Brazil	3	Sequencing and PAGE
9	*D. melanogaster*	Tangará da Serra; (MT); Brazil	3	Sequencing and PAGE
10	*D. melanogaster*	Jari; (RS); Brazil	3	Sequencing and PAGE
11	*D. melanogaster*	Serra da Capivara; (PI); Brazil	3	Sequencing
12	*D. melanogaster*	Manaus; (AM); Brazil	3	Sequencing and PAGE
13	*D. melanogaster*	Fernando de Noronha; (PE); Brazil	3	Sequencing
14	*D. melanogaster*	Fernando de Noronha; (PE); Brazil	3	Sequencing
15	*D. melanogaster*	Recife; (PE); Brazil	3	Sequencing
16	*D. melanogaster*	Ilha Governador; (RJ); Brazil	3	Sequencing
17	*D. melanogaster*	Santa Teresa; (RJ); Brazil	3	Sequencing
18	*D. melanogaster*	Venezuela	3	Sequencing
19	*D. melanogaster*	Vienna; Austria	3	Sequencing and PAGE
20	*D. melanogaster*	Athens; (Attica); Greece	3	Sequencing and PAGE
21	*D. melanogaster*	Prunay; (Marne); France	5	Sequencing and PAGE
22	*D. melanogaster*	La Broche; (Upper Normandy); France	5	Sequencing and PAGE
23	*D. simulans*	Eldorado do Sul; (RS); Brazil	3	PAGE
24	*D. simulans*	Farrapos; (Río Negro); Uruguay	3	PAGE
25	*D. simulans*	Fernando de Noronha; (PE); Brazil	3	PAGE
26	*D. simulans*	Santa Maria; (RS); Brazil	3	PAGE
27	*D. simulans*	Lima; (Lima); Peru	3	PAGE
28	*D. simulans*	Havaí; (HI); United States	3	PAGE
29	*D. simulans*	Rio de Janeiro; (RJ); Brazil	3	PAGE

TPE Repeats: Repeats of TPE found in populations; Method used for obtaining the number of repetitions of TPE: Result obtained by 7% Polyacrylamide Gel Electrophoresis and/or Sanger Sequencing.

The polymerase chain reaction (PCR) was performed under the following conditions: 35 cycles of 15 s at 94°C, 15 s at 53.5°C, and 15 s at 72°C, using the primers TPEf −5'TGCATTTCACAAATTCAAGTCC, TPEr −5'CTTCCCAAAGCCAGTA. These primers were designed from the sequence of the canonical *hobo* element (GenBank access number X04705), using the Primer3 software. This primer pair amplified the fragment of approximately 240 bp over-passing the TPE region.

The products obtained by the PCR reaction were precipitated in 13% polyethylene glycol (PEG8000) and NaCl (1.6 M) and then sequenced directly using a MegaBACE 500 automated sequencer and the DYEnamic ET® kit (Amersham) according to the manufacturer's protocol.

The sizes of PCR fragments were also estimated by non-denaturing 7% PAGE performed according to the protocol described by Sanguinetti*et al.* (1994). These gels measured 21x23 cm with a thickness of 2 mm and electrophoresis was done at 50V for 12 h. The DNA bands were detected using silver staining following the protocol described by Bassam and Gresshoff (2007).

For 22 populations of *D. melanogaster* we sequenced the PCR product containing the TPE region. This showed that all South American populations had three repeats of TPE, while two European populations, Prunay and La Broche, had five. The other two European populations had three repeats (Table 1). Eleven of the populations that had the TPE region sequenced were also analyzed by PAGE. Two Brazilian populations that were analyzed by PAGE only showed the band characteristic of three TPE repeats (Figure 1). These data suggest that the South American populations of *D. melanogaster* were monomorphic for three TPE repeats in*hobo* transposase. In addition, Table 1 shows the origins (collection sites) of the studied populations, the number of TPE repetitions and methods that were used for determining the number of repetitions. The geographic distribution of the South American population sampled is depicted in Figure 2.

**Figure 1:**
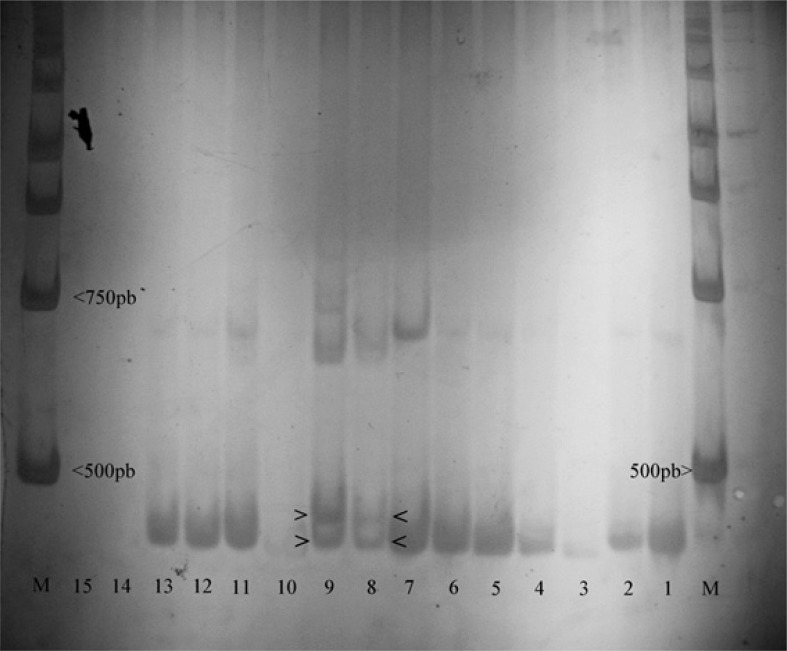
Polyacrylamide gel photo showing the differences in migration patterns of bands of European and Brazilian populations of *D. melanogaster*. (M) markers; (< 500 bp) marker band representing sequence of 500 bp; (< 750pb) marker band representing sequence 750pb; (>) Marking two bands presented by the two European populations, representing regions with polymorphism in the TPE region; (15) Application of negative sample; (14) empty well; (13) Jari, Rio Grande do Sul; (12) Athens, Greece; (11) Vienna, Austria; (10) empty well; (9) Prunay, France; (8) La Broche, France; (7) Erechim, Rio Grande do Sul; (6) Manaus, Amazonas; (5) Porto Alegre, Rio Grande do Sul; (4) Tangara da Serra, Mato Groso; (3) São José do Rio Preto, Sao Paulo, collection point two; (2) São José do Rio Preto, Sao Paulo, collection point 3; (1) São Jose do Rio Preto, Sao Paulo, collection point 4.

**Figure 2:**
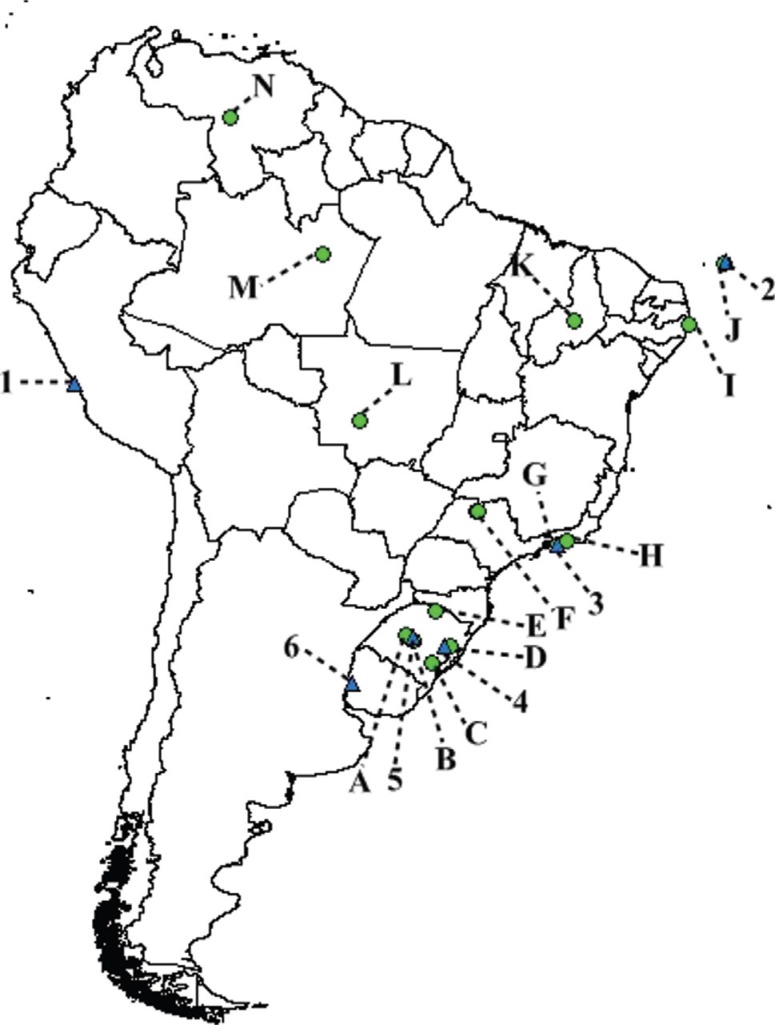
Map of South America with the sampling points of the populations of*D. melanogaster* (green circles, represented by letters) and*D. simulans* (blue triangles, represented by numbers), except for the population from Hawaii. (1) Lima, Peru; (2) Fernando de Noronha, Brazil; (3) Rio de Janeiro, Brazil; (4) Eldorado do Sul, Brazil; (5) Santa Maria, Brazil; (6) Farrapos; Uruguay; (A) Jari, Brazil; (B) Santa Maria, Brazil; (C) Rio Grande, Brazil; (D) Porto Alegre, Brazil; (E) Erechim, Brazil; (F) São José do Rio Preto, Brazil; (G) Ilha do Governador, Brazil; (H) Rio de Janeiro, Brazil; (I) Recife, Brazil; (J) Fernando de Noronha, Brazil; (K) Serra da Capivara, Brazil; (L) Tangará da Serra, Brazil; (M) \Manaus, Brazil; (N) Santa Rosa, Venezuela.

Seven populations of *D. simulans* were analyzed by PAGE electrophoresis and all showed only the band corresponding to three TPE repetitions (Table 1). The populations of D*. simulans* were similar in their band patterns with Brazilian populations of*D. melanogaster* and showed no polymorphism. The geographic distribution of these studied populations can also be found in Figure 2.

As proposed by Periquet *et al.*(1989), American populations of *D. melanogaster* did not contain the *hobo* element before the 1950s, being characterized as strains E (empty). Thereafter, the element was found in American populations and these were characterized as H (*hobo*) strains. Bonnivard *et al.* (2000) and Periquet *et al.* (1989) argued that European populations began to present the *hobo* element since the 1960s, proposing a direction for the invasion of *hobo* element. Periquet *et al.* (1989) suggested that the invasion of the *hobo* element began in America, and from it, the populations carrying the *hobo* element spread all over the world. This element contained three TPE repeats and is commonly found around the world. Bonnivard *et al.* (2000, 2002) added to the evolutionary history of the invasive *hobo* element of *D. melanogaster* the hypothesis of a subsequent invasion of the element containing five or seven TPE repeats. They found polymorphic populations in three regions, central Europe, southern Africa and western South America. In North America, Bonnivard*et al.* (2000) studied 13 populations and found only 3 repeats in nine populations and 3 and 5 repeats in other four populations. From this, the authors suggested that the possible origin of this invasion happened in South America, since in North Africa only populations containing three regions were found. However, North Africa is the geographic region that promotes a connection between the European continent and southern Africa, thus the authors excluded these two regions as possible origins of invasion, suggesting that South America would be the region of origin for the second invasion.

Our results argue against the supposition for the origin of the second invasion, since, like the region intermediate between Europe and southern Africa, our study area, in the East of America also had no polymorphism, hence excluding South America as a possible area of origin for the second invasion.

Thus, of the three regions containing polymorphisms, none is a candidate for the origin of the invasion. From this standoff we suggest that the second invasion of the*hobo* element may not have occurred in South America or in equatorial Africa, and the polymorphism found in these regions may be due to new mutations occurring in these regions followed by selection or genetic drift.

Souames *et al.* (2003a) analyzed the mutation rate of TPE repetitions of the *hobo* element during 20 generations of *D. melanogaster* and found that the mutation rate in this region was a hundred times higher when compared to other regions of neutral microsatellite genome of *D. melanogaster* . Thus, we suggest that the polymorphism found in western South America and southern Africa could be the result of mutations that were selected or suffered genetic drift, because none of these places has the potential to be the geographic region of origin of the supposed second invasion.

Unlike these regions, Central Europe has certain peculiarities not shared by South America and Africa. In Europe, the TPE polymorphism frequency is much higher than in any other region and, in addition, presents many kinds of polymorphisms with copies ranging from three repeats and others containing up to ten repetitions (Bonnivard *et al.*, 2002). Thus it is possible that this polymorphism is a local one generated by new mutations.

The monomorphism found in *D. melanogaster* in the TPE regions was shared by *D. simulans* . So far, the TPE region of *D. simulans*has not been widely studied, but for this current study it is important to discuss the supposed donor of the *hobo* element in *D. melanogaster*, the sister species, *D. simulans* . Boussy and Daniels (1991) indicated a direction of the origin of the*hobo* element in *D. melanogaster*, indicating that the donor species of the element would be the sister species *D. simulans*, and this direction of invasion is supported for two reasons:*hobo* would be present in *D. simulans* before*D. melanogaster* and the high similarity of the element between the two species. Periquet *et al.*(1989) and Bonnivard *et al.*(2000, 2002) argued that the invasion of the *hobo* element in populations of *D. melanogaster*happened in the 1950s in Central America and the *hobo* element had three repetitions of TPE, so for *D. simulans* to be the donor element it would also have to present the *hobo* element with three repetitions. Our results supported the hypothesis proposed by Boussy and Daniels (1991) of *D. simulans* as the donor of the*hobo* element to *D. melanogaster* because all populations studied had three replications of TPE (Figure 2 and Table 1). In this way we can infer with more clarity that *D. simulans* was possibly the donor of the*hobo* element to *D. melanogaster*.

The TPE region has been used by the scientific community because it contains information about the history of the *hobo* element and its dynamics. Based on the TPE repeats, the evolutionary scenario of the *hobo* element was proposed and their history was better understood (Calvi *et al.*, 1991; Bazin and Higuet, 1996; Bonnivard *et al.*, 2000, 2002;). Our results are in accordance with the hypothesis of multiple origins for the second hobo invasion, as suggested by Bonnivard *et al.*(2000).
